# Aberrant ROS Served as an Acquired Vulnerability of Cisplatin-Resistant Lung Cancer

**DOI:** 10.1155/2022/1112987

**Published:** 2022-06-20

**Authors:** Qian Xin, Qinghong Ji, Ying Zhang, Weihong Ma, Baoqing Tian, Yanli Liu, Yunsong Chen, Fei Wang, Ran Zhang, Xingwu Wang, Jupeng Yuan

**Affiliations:** ^1^Central Laboratory, The Second Hospital, Cheeloo College of Medicine, Shandong University, Jinan, Shandong 250033, China; ^2^Department of Obstetrics, The Second Hospital, Cheeloo College of Medicine, Shandong University, Jinan, Shandong 250033, China; ^3^Department of Respiratory Medicine, The Second Hospital, Cheeloo College of Medicine, Shandong University, Jinan, Shandong 250033, China; ^4^Shandong Provincial Key Laboratory of Radiation Oncology, Cancer Research Center, Shandong Cancer Hospital and Institute, Shandong First Medical University and Shandong Academy of Medical Sciences, Jinan, Shandong 250117, China; ^5^Key Laboratory for Experimental Teratology of the Ministry of Education, Shandong University, Jinan, Shandong 250033, China

## Abstract

Lung cancer has become a global health issue in recent decades. Approximately 80-85% of cases are non-small-cell lung cancer (NSCLC). Despite the high rate of resistance, cisplatin-base chemotherapy is still the main treatment for NSCLC patients. Thus, overcoming cisplatin resistance is urgently needed in NSCLC therapy. In this study, we identify NADPH metabolism and reactive oxygen species (ROS) levels as the main causes accounting for cisplatin resistance. Based on a small panel consisting of common chemotherapy drugs or compounds, APR-246 is proved to be an effective compound targeting cisplatin-resistant NSCLC cells. APR-246 specially inhibits proliferation and colony formation of cisplatin-resistant cells. In details, APR-246 can significantly cause G0/G1 accumulation and S phase arrest of cisplatin resistant cells and gives rise to severe mitochondria dysfunction as well as elevated apoptosis. Further study proves that it is the aberrant ROS levels as well as NRF2/SLC7A11/GSH axis dysfunction accounting for the specific antitumor effects of APR-246. Scavenging ROS with N-acetylcysteine (NAC) disrupts the inhibitory effect of APR-246 on cisplatin-resistant cells. Mechanistically, NRF2 is specifically degraded by the proteasome following its own ubiquitylation in APR-246-treated cisplatin-resistant cells, which in turn decreases NRF2/SLC7A11/GSH axis activity. Our study provides new insights into the biology driving cisplatin resistance of lung cancer and highlights APR-246 as a potential therapeutic reagent for overcoming cisplatin resistance.

## 1. Introduction

Cancer is a major public health issue and has led to a remarkable global burden of overall diseases in recent decades [1, 2]. As the most common type of malignant tumors in the world, lung cancer contributes to 25% of male and 20% of female cancer-related deaths [1–5]. The cancer report data released by the International Agency for Research on Cancer (IARC) shows that lung cancer is still the leading cause of cancer mortality worldwide in 2020 [6]. Epidemiological data reveals that smoking status, ambient air pollution, genetic susceptibility, as well as difficulties of early diagnosis and insufficient cure in the late stage are responsible for this phenomenon [7]. Currently, surgery, chemotherapy, tyrosine kinase inhibitor- (TKI-) based targeted therapy, and immunotherapy are main therapies of lung cancer [8–10]. Despite the high rate of resistance, platinum-based chemotherapy is still one of the main treatment options for lung cancer patients who are not suitable for surgery, TKI-based targeted therapy, and immunotherapy [9, 11].

As the most commonly used platinum-based chemotherapeutic drug, cisplatin exhibits effective and broad-spectrum antitumor activity against multiple cancers, including bladder cancer, ovarian cancer, breast cancer, testicular cancer, and lung cancer [12, 13]. Numerous studies indicate that the anticancer effect of cisplatin is derived from its ability to cross-link with DNA and subsequently inhibits DNA replication and transcription, followed by activation of apoptosis pathway [14, 15]. Cisplatin is the first chemotherapeutic choice and has been proven to be a very successful reagent in the treatment for NSCLC. However, resistance to cisplatin remains the key issue in tumor treatment and limits its clinical efficacy. Genetic heterogeneity and aberrant gene expression may be the intrinsic factors that cause primary cisplatin primary resistance. Mutations of the *p53* gene can lead to primary cisplatin resistance in ovarian cancer as a consequence of loss of the ability of p53 to transactivate *BAX*, an apoptosis-inducing gene [16]. And the 10, 27, 60, and 70 kDa heat-shock proteins have also been linked to stress response in protein folding and unfolding in primary cisplatin resistance [17]. Besides, DNA methylation, histone modification, chromatin remodeling factors as well as noncoding RNAs are also important factors affecting cisplatin sensitivity [18]. According to effective phases, several mechanisms contributing to cisplatin resistance have been described: (i) pretarget resistance, (ii) on-target resistance, (iii) posttarget resistance, and (iv) off-target resistance [15, 19]. Besides, acquired cisplatin resistance is more complex than primary cisplatin resistance. Therefore, attenuation of the acquired chemoresistance to cisplatin is an important and urgently needed clinical objective.

The p53 tumor suppressor protein plays an important role in the control of tumor cell response to chemotherapy [20]. It is well established that the deletion or mutation of p53 involve in cellular resistance to cisplatin [21]. APR-246 (PRIMA-1^MET^) is a low molecular weight compound, which could restore tumor suppressor activity of mutant p53 and trigger cancer cell apoptosis by upregulating p53 target genes such as *BAX*, *PUMA*, and *NOXA* genes in various cancer types [22]. In addition to binding to p53, APR-246 can also induce formation of reactive oxygen species (ROS) in tumor cells and deplete intracellular glutathione (GSH) concentration [23]. Thus, APR-246 is a potential treatment for drug-resistant tumors. However, the molecular mechanisms underlying APR-246 overcoming drug resistance are not completely clear.

Aberrant ROS are found in cisplatin-resistant cell lines by several research groups, indicating essential roles of ROS in cisplatin resistance [24–27]. Here, we have detected ROS levels in both cisplatin resistance and parental cells and assessed the antitumor activity of APR-246 based on *in vitro* and *in vivo* experiments. Results indicate NADPH metabolism and ROS levels are the main causes accounting for cisplatin resistance. Besides, APR-246 can selectively inhibit proliferation and colony formation of cisplatin-resistant cells instead of parental cells through mitochondria-mediated apoptosis. Mechanistic analysis reveals that nuclear factor erythroid 2-related factor 2 (NRF2) is specifically degraded through ubiquitin proteasome pathway in APR-246-treated cisplatin-resistant cells, which in turn decrease NRF2/SLC7A11/GSH axis activity. In short, our study gives a new insight into the potential mechanisms of cisplatin resistance of lung cancer and shows APR-246 as a promising therapeutic drug again cisplatin resistance.

## 2. Materials and Methods

### 2.1. Cell Culture

NCI-H460 (RRID: CVCL_0459, H460 for short) and H460-Cis cells were purchased from the Shanghai Cell Collection (Chinese Academy of Sciences). A549 (RRID: CVCL_0023) and A549-Cis cells were obtained from the Cell Resource Center, Peking Union Medical College. All these cell lines were cultured in Dulbecco's modified Eagle's medium (DMEM) (Gibco, Grand Island, NY, USA) supplemented with 10% fetal bovine serum (FBS) (Hyclone, Logan, UT, USA). H460-Cis and A549-Cis cells were treated with 2 *μ*g/mL cisplatin to maintain cisplatin resistance character. All human cell lines have been authenticated using short tandem repeat (STR) profiling. Cells were maintained at 37°C in a humidified atmosphere with 5% CO_2_. Cells were regularly tested for mycoplasma and were mycoplasma free as previously reported [28].

### 2.2. Reagents

The compound APR-246 and the proteasome inhibitor MG132 were purchased from MedChemExpress (China). Cycloheximide (CHX), carboplatin, doxorubicin, paclitaxel, and fluorouracil (5-FU) were purchased from MP Biomedicals (France). N-acetyl-L-cysteine (NAC) was bought from Beyotime Biotechnology (Beijing, China). For *in vitro* experiments, 100 mM APR-246 and 750 mM NAC were dissolved in DMSO, and ultrasonic was used for improving its solubility. MG132, CHX, and paclitaxel were dissolved in DMSO to 50 mg/mL, 50 mg/mL, and 100 mg/mL, respectively. Carboplatin (10 mg/mL), doxorubicin (100 mg/mL), and 5-FU (10 mg/mL) were dissolved in water. For *in vivo* experiments, APR-246 was rinsed with sterilized PBS and ultrasonized into clear solutions at concentration 100 mg/mL, and NAC was directly dissolved at 5 mg/mL in the drinking water. All the reagents were stored at -20°C for long-term preservation.

### 2.3. Quantification of ROS Level

ROS levels were detected using 2′,7′-dichlorofluorescin diacetate (DCFDA) cellular ROS detection assay kit (Beyotime Biotechnology, Beijing, China) following manufacturers' protocol. In short, cells were seeded in 6-well plates. Then, cell samples were incubated with 100 *μ*M DCFDA at 37°C for 30 min and analyzed using flow cytometry (BD Biosciences, Mississauga, Ontario). Each experiment was performed in triplicate.

### 2.4. Quantification of NADP^+^/NADPH

The intracellular NADP^+^ and NADPH levels were measured using the NADP^+^/NADPH assay kit (Abcam, Inc., MA, USA). Experiments were performed according to the manufacturer's protocols, and the NADP^+^ and NADPH concentrations were determined colorimetrically based on absorbance at 565 nm. Each experiment was performed in triplicate.

### 2.5. Quantification of GSH and GSSG

Total (GS), oxidized (GSSG), and reduced (GSH) glutathione concentrations were measured using the GSH/GSSG ratio detection assay kit (Abcam, Inc., MA, USA) and a fluorescence microplate reader with excitation and emission wavelengths of 490 and 520 nm, respectively. Each experiment was performed in triplicate.

### 2.6. MTT Assay

Same as before [29], cells at a concentration of 5 × 10^4^ cells/well were seeded in 100 *μ*L culture medium into microplates (tissue culture grade, 96 wells), and the microplates were incubated in cell cultures for 72 h or 96 h at 37°C and 5% CO_2_. After the incubation period, 10 *μ*L of the MTT labeling reagents (final concentration 0.5 mg/mL) was added to each well at preset time points, including 0 h, 24 h, 48 h, and 72 h. Then, we incubated the microplate for 4 h in a humidified atmosphere. And 4 h later, 100 *μ*L of the solubilization solution was added into each well. After hatching in the incubator overnight, we checked for complete solubilization of the purple formazan crystals and measure the absorbance of the samples using a microplate reader. Absorbance of formazan products was measured at 570 nm using a microplate spectrophotometer (Tecan, Mannedorf, Switzerland).

### 2.7. Colony Formation Assay

Briefly, cells were trypsinized, counted, and replated in six-well plate to allow formation of macroscopic colonies. After 7 to 14 days, the cell colonies were fixed with methanol and stained with crystal violet. Stained colonies containing at least 50 cells in size were counted.

### 2.8. Apoptosis and Cell Cycle Assay

In apoptosis assay, cells were classified into different groups, according to combination treatment of APR-246 and/or NAC. Then, nonadherent and adherent cells were collected at 48 hours after treatment. Cell samples were stained with Alexa Fluor 488 Annexin V/dead cell apoptosis kit (Roche, USA). The percentage of apoptotic cells was determined by Calibur flow cytometer (FCM) (BD Biosciences, Mississauga, USA). As for cell cycle analyses, cells were dyed with propidium iodide (Beyotime, China).

### 2.9. Reverse-Transcription PCR and Real-Time PCR Assay

RNA extraction and RT-PCR were performed as described previously [30]. Total RNA was extracted with TRIZOL reagent following the manufacturer's instructions (Invitrogen, Carlsbad, CA, USA). Potential contaminating genomic DNA was removed by RNase-free DNase treatment (Promega, Madison, WI, USA). cDNA was prepared with the MMLV Reverse Transcriptase (Takara, Japan). Relative quantitation used the ABI QuantStudio6Flex System (Thermo Fisher, USA) and determined by 2^−ΔΔct^ method. The primer sequences of real-time PCR are shown in the Supplemental Table 1. Each experiment was performed in triplicate.

### 2.10. Western Blotting

Western blotting experiment was performed as described previously [31]. Equal amounts of protein extracts were separated by SDS-polyacrylamide gel, transferred to a polyvinyl difluoride membrane (PVDF) membrane (Roche; Roche Diagnostics, Basel, Switzerland) at 37°C for 1 h, and followed by incubation with specific primary antibodies overnight at 4°C. Membranes were incubated with horseradish peroxidase- (HRP-) conjugated secondary antibodies at room temperature for 1 hour and detected using the electrochemiluminescence (ECL) kit (Thermo Scientific, Rockford, IL, USA). The detailed sources and other information of antibodies were listed in the supplemental materials (Supplemental Table 2).

### 2.11. CHX-Mediated Chase Assay

To assess protein stability, cells were treated with 200 *μ*g/mL CHX to block de novo protein synthesis. At the present time points, cell lysates were prepared and then subjected to immunoblotting.

### 2.12. Xenograft

Female BALB/c nude mice (4~5 weeks of age) were maintained in specific pathogen-free conditions from Shandong Cancer Hospital and Institute. For xenografts, about 6.0 × 10^6^ viable cells in 100 *μ*L PBS were injected subcutaneously into the nude mice. Five animals per group were used in each experiment. APR-246 (50 mg/kg or 100 mg/kg) was injected i.p. at preset timepoint. NAC (5 mg/mL) was given in the drinking water for the length of the experiment until sacrifice. Tumor sizes were measured every 4 days, and mice were euthanized when tumors reached 1.5 cm in diameter. The volume was calculated according to the formula: 1/2 × length × squared width. All studies were approved by the Animal Care Committee of Shandong Cancer Hospital.

### 2.13. Statistical Analysis

Data from the two groups were evaluated statistically by a two-tailed unpaired *t*-test using GraphPad Prism version 7.0 (GraphPad, San Diego, CA, USA) (Serial number: GPS-0320559-L###-#####). In these analyses, *p* values less than 0.05 were considered significant (^∗^*p* < 0.05, ^∗∗^*p* < 0.01, ^∗∗∗^*p* < 0.001).

## 3. Results

### 3.1. Decreased NADPH Oxidase-Mediated ROS Accounts for Cisplatin Resistance of Non-Small-Cell Lung Cancer (NSCLC)

**Figure 1:**
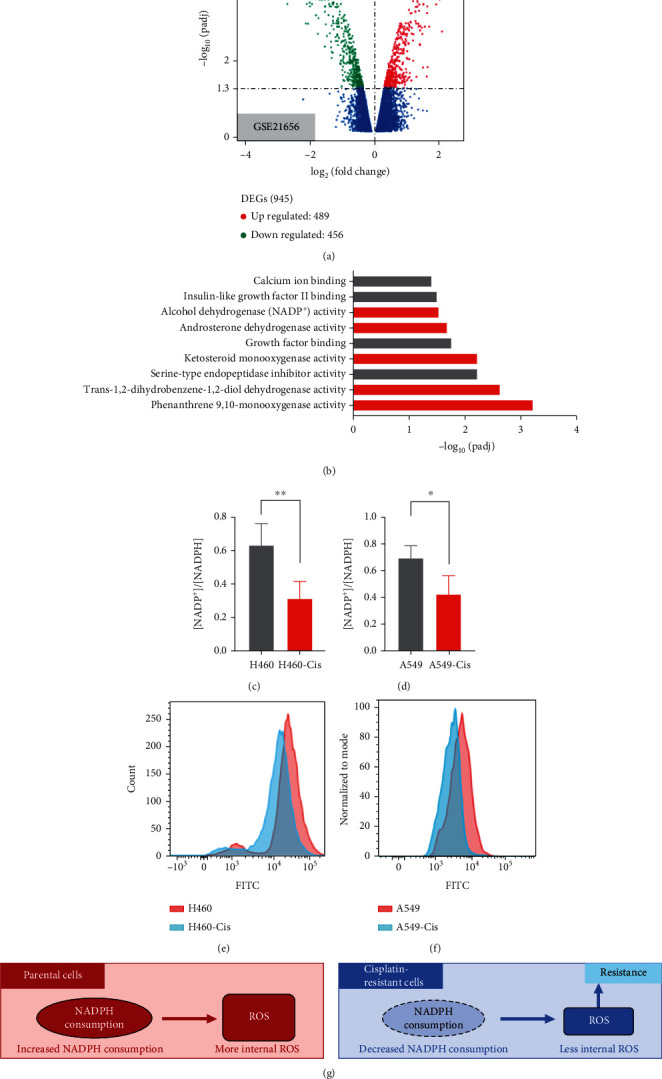
Role of NADPH oxidase-mediated ROS in cisplatin resistance of NSCLC. (a) Differentially expressed genes in cisplatin-resistant H460 cells and its parental cells (GSE21656). *p* values for significance are set at 0.05. Red dots stand for upregulated genes, while downregulated genes are shown in green dots. Unchanged genes are colored in blue. (b) Signaling pathways related to cisplatin resistance are analyzed by GO methods. Metabolic pathways of NADPH are highlighted in red. (c, d) Quantification of NADP^+^ and NADPH in H460-Cis and A549-Cis as well as their parental cells. (e, f) ROS levels of H460-Cis and A549-Cis as well as their parental cells are detected by FCM. (g) Mechanism diagram presenting important role of NADPH oxidase-mediated ROS in cisplatin resistance of NSCLC. Error bars represent SD of three independent experiments. ^∗^*p* < 0.05; ^∗∗^*p* < 0.01; ^∗∗∗^*p* < 0.001 (two-tailed unpaired *t*-test).

Cisplatin resistance is a common outcome of NSCLC patients treated with cisplatin [9, 11]. Several mechanisms revealing cisplatin resistance are proposed by different research teams [15, 19]. In this study, we first analyzed differentially expressed genes based on high-throughput sequencing results published elsewhere between H460-Cis cells and parental cells (GSE21656) [32]. Differentially expressed genes were screened out through standard pipelines, and results were displayed through volcano plot. A total of 489 genes were upregulated, and 456 genes were downregulated in H460-Cis cells compared to parental cells (Figure 1(a)). Related signaling pathways were then analyzed by Gene Ontology (GO) analysis method based on different expressed genes in H460-Cis cells. Among top-ranked pathways, five NADPH metabolism pathways were identified, including monooxygenase activity or dehydrogenase activity of different chemicals (Figure 1(b)). Bioinformatics analysis results suggested that NADPH metabolism might play critical roles in cisplatin resistance. Thus, we evaluated that NADPH metabolism levels in both H460-Cis and A549-Cis cells as well as their parental cells (cisplatin-resistant features of these cell lines were provided in Figure S1A-S1F). Indeed, our result showed that [NADP+]/[NADPH] ratio was significantly decreased in cisplatin H460-Cis and A549-Cis cells compared to their parental cells (Figures 1(c) and 1(d)). As we all know, NADPH serves as the ultimate donor of reductive power for the large majority of ROS-detoxifying enzymes. Consistent with results of NADP+/NADPH quantification, ROS levels of H460-Cis and A549-Cis cells were much lower than that in their parental cells (Figures 1(e) and 1(f)). These results indicated that decreased NADPH oxidase-mediated ROS might account for cisplatin resistance of NSCLC (Figure 1(g)).

### 3.2. Effects of Common Chemical Drugs on Cisplatin-Resistant NSCLC Cells

**Figure 2:**
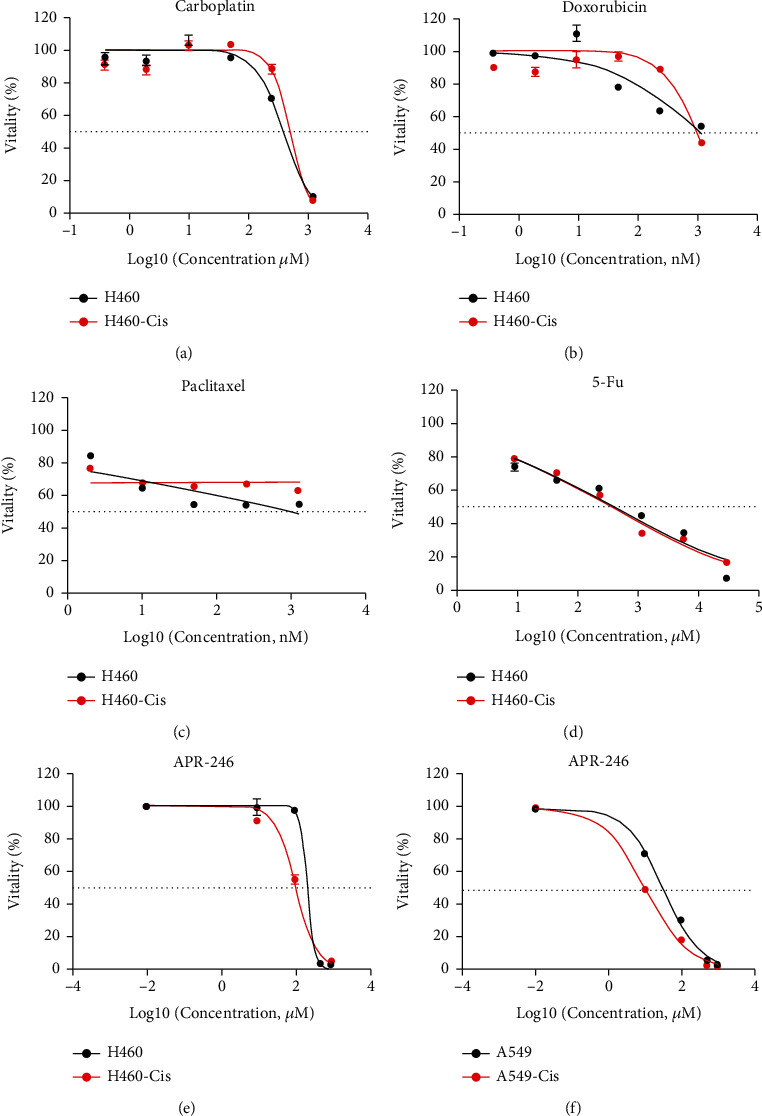
Antitumor effects of APR-246 in both H460 and H460-Cis cells. (a–e) Dose-response curves of carboplatin (a), doxorubicin (b), paclitaxel (c), and 5-Fu (d) as well as APR-246 (e) in both H460 and H460-Cis cells. (f) Dose-response curves of APR-246 in A549 and A549-Cis cells.

In order to find out novel reagents overcoming cisplatin resistance, we evaluated the effects of commonly used chemical drugs or compounds by measuring survival fractions of both H460-Cis and A549-Cis cells as well as their parental cells in order to identify chemicals specifically killing cisplatin-resistant cells. Carboplatin, doxorubicin, paclitaxel, and 5-fluorouracil (5-Fu), as well as APR-246, a commonly used p53 allosteric compounds, were enrolled in this screen design. Consistent with our expectation, H460-Cis cells were also resistant to common chemical drugs including carboplatin, doxorubicin, and paclitaxel (Figures 2(a)–2(c)). There was no difference in IC50 values for 5-Fu between H460 and H460-Cis cell lines (Figure 2(d)). Only APR-246 specially killed H460-Cis and A549-Cis cells (Figures 2(e) and 2(f)). Despite low killing efficiencies of APR-246 at low concentration, there were obvious separations between survival fractions of APR-246 treatment H460 and H460-Cis cells (Figure S2A). Besides, the killing effects of APR-246 in H460-Cis cells were in dose-dependent and time-dependent manners (Figure S2B-S2C).

### 3.3. APR-246 Specifically Inhibited Proliferation of Cisplatin-Resistant Cells through Disrupting Cell Cycle

**Figure 3:**
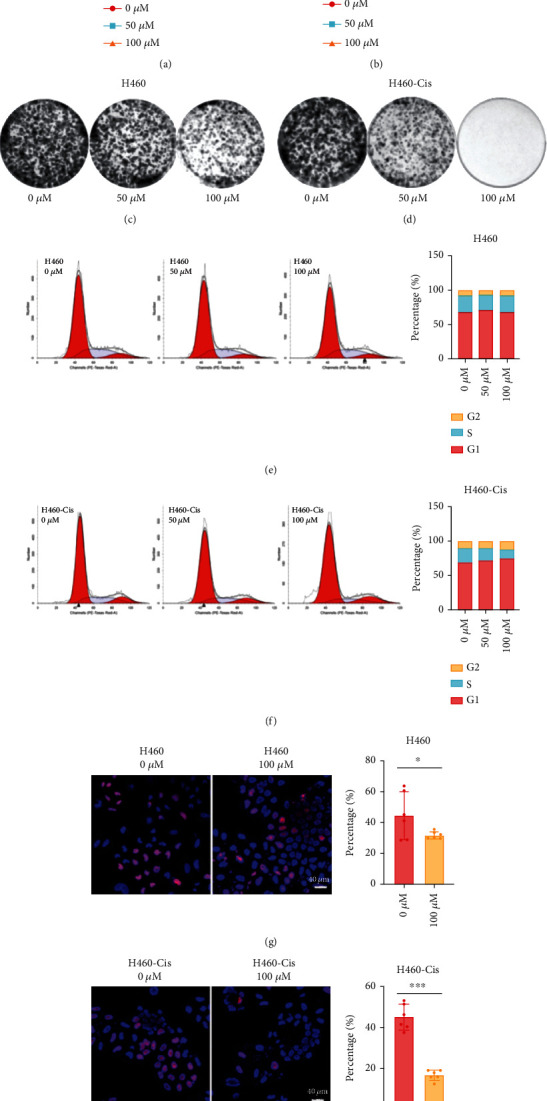
Dysregulation of cell cycle caused by APR-246 in H460-Cis cells. (a, b) Growth curves of H460 and H460-Cis cells treated with APR-246 or not. (c, d) Colony formation assays of H460 and H460-Cis cells treated with APR-246 or not. (e, f) Cell cycle distribution of H460 and H460-Cis cells treated with APR-246 or not. (g, h) Results of EdU incorporation assay detected in APR-246 treating H460 and H460-Cis cells. Error bars represent SD of three independent experiments. ^∗^*p* < 0.05; ^∗∗^*p* < 0.01; ^∗∗∗^*p* < 0.001 (two-tailed unpaired *t*-test).

As shown in Figure 3(b) and Figure S3B, APR-246 could specially inhibit proliferation of H460-Cis and A549-Cis cells at both internal and high dose. However, there was no differences among these different groups in their parental cells (Figure 3(a) and Figure S3A). The results of colony formation assay demonstrated that APR-246 could inhibit both cisplatin-resistant and parental cells. It is worth noting that APR-246 displayed more significant inhibitory effects in cisplatin cells than parental cells (Figures 3(c) and 3(d) and Figure S3C-S3D). Results of cell cycle indicated that the cisplatin-resistant cells were accumulated in G0/G1 phase after APR-246 treatment (Figures 3(e) and 3(f) and Figure S3E-S3F). Besides, cell cycle results suggested that the percentage of S phase was much lower in cisplatin-resistant cells compared to untreated groups (Figure 3(f) and Figure S3F). EdU incorporation assay was also performed, and EdU incorporation rate of APR-246-treated group was much lower than untreated groups in cisplatin-resistant cells. Although changes of EdU incorporation rate were also detected between APR-246-treated and APR-246-untreated H460 cells, the inhibitory effects were significantly weaker than H460-Cis cells (Figures 3(g) and 3(h) and Figure S3G-S3H).

### 3.4. APR-246 Induced Mitochondrial-Mediated Cell Apoptosis in Cisplatin-Resistant Cells

**Figure 4:**
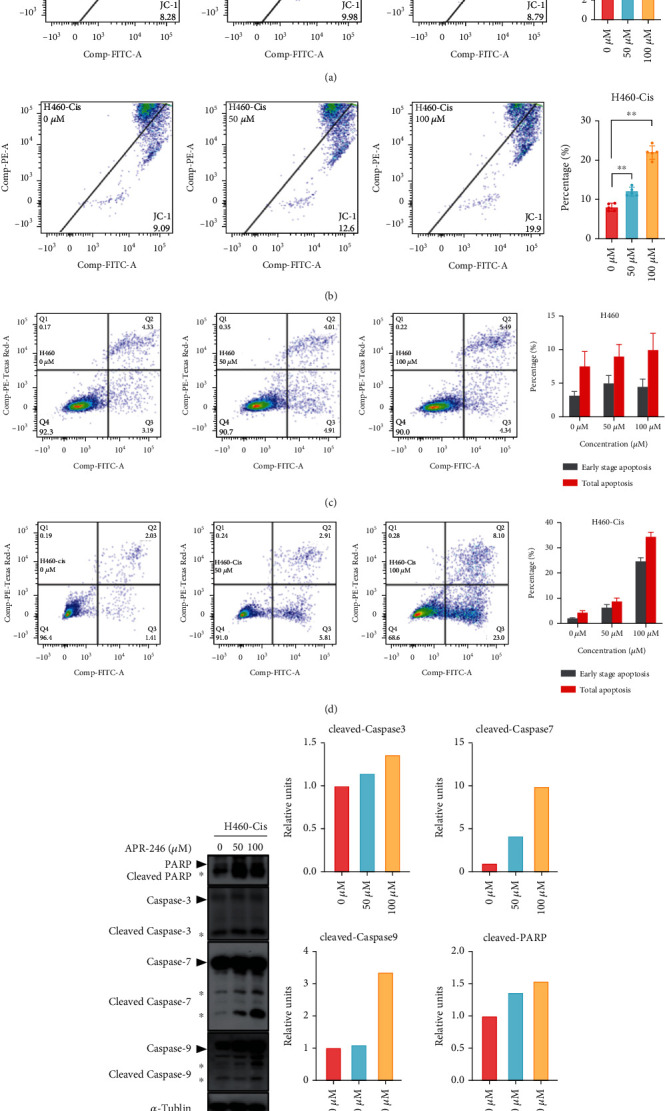
APR-246 leads to mitochondria-mediated apoptosis in H460-Cis cells. (a, b) Mitochondrial membrane potentials of H460 and H460-Cis cells treated with APR-246 are detected by JC-1 assay. Representative results are shown on the left, and statistical analysis is on the right. (c, d) Apoptosis results of H460 and H460-Cis cells treated with APR-246 or not. Representative results are shown on the left, and statistical analysis is on the right. (e) Western blotting results of PARP, Caspase-3, Caspase-7, and Caspase-9 as well as their cleaved forms. (f) Protein levels of cleaved PARP, cleaved Caspase-3, cleaved Caspase-7, and cleaved Caspase-9 are quantified by densitometric analysis using Quantity One software. Error bars represent SD of three independent experiments. ^∗^*p* < 0.05; ^∗∗^*p* < 0.01; ^∗∗∗^*p* < 0.001 (two-tailed unpaired *t*-test).

Apoptosis mediated by mitochondria dysfunction was a main outcome of cancer cells under treatment of chemical drugs [33–35]. A distinctive feature of the early stages of programmed cell death is the disruption of active mitochondria. In this part, JC-1 assay was performed to detect early changes in mitochondria by flow cytometry. As shown in Figures 4(a) and 4(b) and Figure S4A-S4B, only cisplatin-resistant cells manifested severe mitochondria dysfunctions in dose-dependent manners after APR-246 administration. Apoptosis was also examined. Consistent with changes of mitochondria, APR-246 treatment resulted more apoptosis only in cisplatin-resistant cells (Figures 4(c) and 4(d) and Figure S4C-S4D). Protein levels of cleaved PARP, cleaved Caspase-3, cleaved Caspase-7, and cleaved Caspase-9 were significantly increased after treatment of APR-246 in cisplatin-resistant cells (Figures 4(e) and 4(f)). All these results confirmed that APR-246 could lead to mitochondrial-mediated apoptosis in cisplatin-resistant cells.

### 3.5. APR-246 Disrupted NRF2/SLC7A11/GSH Axis through Mediating NRF-2 Degradation

**Figure 5:**
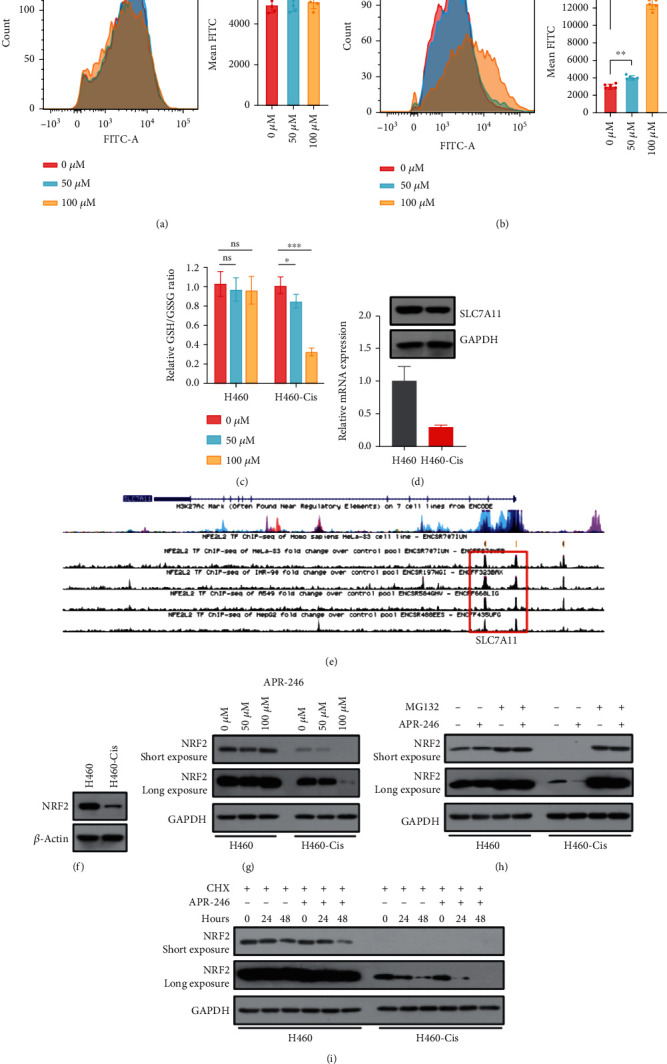
APR-246 promotes NRF2 degradation and alerts NRF2/SLC7A11/GSH axis. (a, b) Detection of ROS levels in APR-246 treating H460 and H460-Cis cells by FCM. Representative results are shown on the left, and statistical analysis is shown on the right. (c) GSH and GSSG levels detected in both H460 and H460-Cis cells treated with APR-246 or not. (d) Protein and mRNA levels of SLC7A11 in H460 and H460-Cis cells. The upper panel stands for Western blotting results, while real-time PCR results are shown in the lower panel. (e) ChIP-sequencing results of NRF2 and H3K27Ac are shown by UCSC browser. Binding peaks of NRF2 at SLC7A11's promoter is highlighted within the red box. (f) Protein levels of NRF2 in H460 and H460-Cis cells are detected by Western blotting. (g) Western blotting results of NRF2 in both APR-246 treating H460-Cis and parental cells. (h) Western blotting of whole cell lysates of the indicated cells treated with or without 30 *μ*M MG132 for 24 h. (i) APR-246 treating H460-Cis and parental cells are administrated with 50 *μ*g/mL cycloheximide, harvested at the indicated time points, and then subjected to Western blotting. Error bars represent SD of three independent experiments. ^∗^*p* < 0.05; ^∗∗^*p* < 0.01; ^∗∗∗^*p* < 0.001 (two-tailed unpaired *t*-test).

In the first part of this study, we showed that ROS levels of cisplatin-resistant cells were much lower than parental cells (Figure 1(d)). However, the changes of ROS after APR-246 treatment were unclear. So, we examined ROS changes in both APR-246 treating cisplatin-resistant and parental cells. We found there were no changes among APR-246 treating and control groups of parental cells (Figure 5(a) and Figure S5A). However, APR-246 did aggravate ROS levels significantly in cisplatin-resistant cells, especially at high dose (Figure 5(b) and Figure S5B). Considering important roles of APR-246 in mediating GSH metabolism, GSH/GSSG ratios were also examined. Consistent with previous findings, APR-246 resulted in apparent decrease of GSH/GSSG ratios only in cisplatin-resistant cells (Figure 5(c)). In order to verify molecular mechanisms of this phenomenon, SLC7A11, the main regulator of GSH metabolism, was detected at both mRNA and protein levels (Figure 5(d)). As shown in Figure 5(d), the amount of SLC7A11 in H460-Cis cells was much lower than that of H460 cells at both transcript and protein levels, indicting SLC7A11 might be disrupted by APR-246 at transcriptional level. The transcription factor NRF2 is the master regulator of neutralizing cellular ROS and restoring redox balance [36]. Indeed, there was obvious NRF2-binding peaks at the promoter of *SLC7A11* based on experiments collected by ENCODE database (Figure 5(e)). It indicated that NRF2 might be the core transcription factor of SLC7A11. Consistent with expression differences of SLC7A11 between H460-Cis and H460 cells, H460-Cis cells possessed less amount of NRF2 compared to H460 cells (Figure 5(f)). Moreover, NRF2 was downregulated after APR-246 treatment in H460-Cis cells, while NRF2 was slightly changed in H460 cells (Figure 5(g)). To explore detailed mechanism of NRF2 downregulation, we detected mRNA levels of *NFE2L2* (NRF2 coding gene) after APR-246 treatment at different dose. No changes of NRF2 mRNA were detected (Figure S5C). Despite slight changes of NRF2 protein levels in H460 cells treated with APR-246 or not, MG132 could induce obvious accumulation of NRF2. And the downregulation of NRF2 caused by APR-246 treatment of H460-Cis could also be totally rescued by MG132 (Figure 5(h)). It revealed that 26S proteasome-mediated degradation of NRF2 accounted to its decrease in APR-246-treated H460-Cis cells. To further illustrate whether APR-246 regulates NRF2 degradation, we examined NRF2 expression in H460-Cis and H460 cells incubated in the presence of the protein synthesis inhibitor cycloheximide (CHX) with or without APR-246. We found that the NRF2 protein levels decreased much faster in H460-Cis cells treated with APR-246 than those treated with DMSO (Figure 5(i)). However, the degradation rates were unchanged in H460 cells treated with APR-246 or not. All these results indicated that APR-246 could disrupt ROS-related NRF2/SLC7A11/GSH axis through mediating NRF-2 degradation only in cisplatin-resistant cells.

### 3.6. ROS Accounted for Antitumor Activity of APR-246 in Cisplatin-Resistant Cells

**Figure 6:**
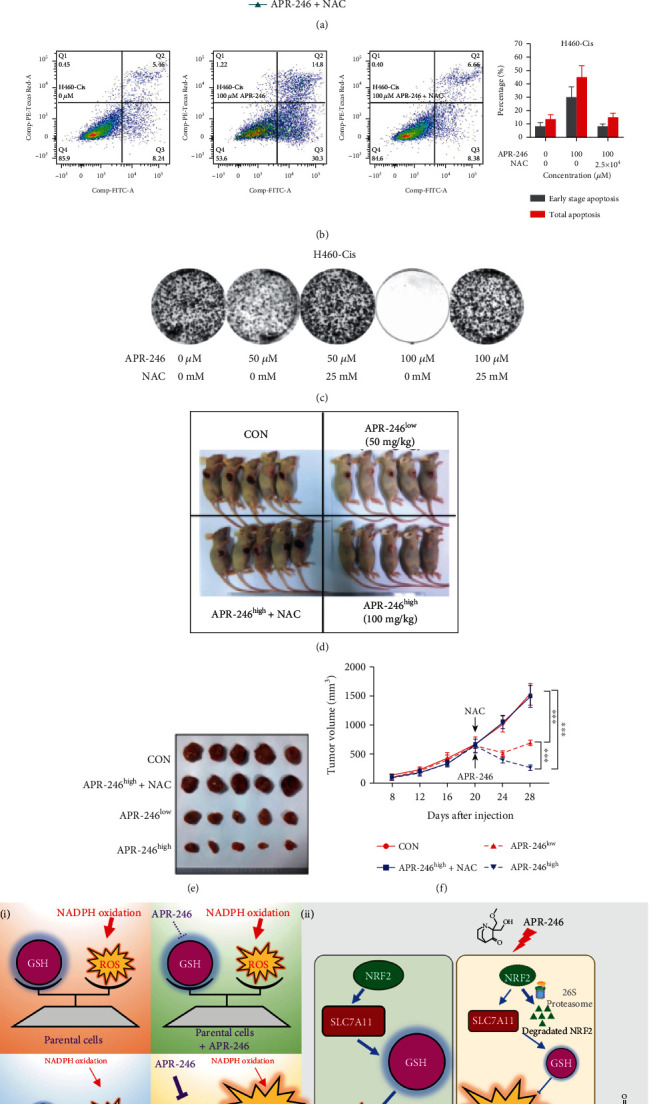
NAC disrupts antitumor effects of APR-246. (a) Proliferation of H460-Cis cells treated with APR-246 or/and NAC. (b) Apoptosis levels of H460-Cis cells treated with APR-246 or/and NAC are detected by FCM. The right panel stands for statistical analysis results. (c) Colony formation efficiency of H460-Cis cells treated with APR-246 or/and NAC. (d–f) Xenograft of H460-Cis cells treated with APR-246 or/and NAC. Photos of tumors are shown in (d, e). Tumor growth curves are provided in (f). Each group contained five mice. Error bars represent the SEM. APR-246 (50 mg/kg or 100 mg/kg) was injected i.p. at preset timepoint. NAC (5 mg/mL) was given in the drinking water uninterruptedly until sacrifice. (g) Graphical representation of the relationship between APR-246 and cisplatin resistance of NSCLC. Error bars represent the SD of three independent experiments. ^∗^*p* < 0.05; ^∗∗^*p* < 0.01; ^∗∗∗^*p* < 0.001 (two-tailed unpaired *t*-test).

To further verify whether APR-246 specially inhibits cisplatin-resistant cells through interfering ROS levels, we used N-acetylcysteine (NAC), a common ROS scavenger, to perform functional rescue experiments. As shown in Figure 6(a) and Figure S6A, NAC could disrupt inhibited proliferation of cisplatin-resistant cells caused by APR-246. Further analysis of apoptosis also confirmed this result. Despite APR-246 leads severe apoptosis, NAC could significantly reduce apoptosis rates to normal levels in APR-246 treating cisplatin-resistant cells (Figure 6(b) and Figure S6B). Moreover, colony formation results also demonstrated that NAC could totally eliminate inhibiting effects of APR-246 in both internal and high-dose group (Figure 6(c) and Figure S6C). We next evaluated the *in vivo* role of APR-246 using H460-Cis xenografts. Consistent with *in vitro* results above, growth of xenografts treated with APR-246 (both low and high dose) were severely impaired compared to the control group (*p* < 0.001), while tumors of xenografts treating with both APR-246 (high concentration) and NAC were much similar to those of controls (Figures 6(d)–6(f) and Figure S6D).

## 4. Discussion

Inherent and acquired cisplatin resistance reduces the effectiveness of this reagent in the management of NSCLC [9, 11, 37]. Thus, understanding the molecular mechanisms underlying this process is meaningful for the development of novel reagents to enhance the sensitivity of cisplatin. In this study, we screened compounds that might overcome cisplatin resistance based on a small panel. APR-246 was chosen due to its lower IC50 and higher inhibiting efficiency on cisplatin resistant cells. Further study demonstrated that cell cycle and apoptosis were dysregulated upon APR-246 treatment in cisplatin-resistant cells. Mechanistically, NRF2 is specifically degraded in APR-246-treated cisplatin-resistant cells through ubiquitin-mediated proteolysis, which in turn decrease NRF2/SLC7A11/GSH axis activity. Moreover, this axis could be totally impaired by ROS scavenger (NAC) (Figure 6(g)).

APR-246, also known as PRIMA-1^MET^, is a small organic molecule that has been shown to restore tumor-suppressor function primarily to mutant p53 and also to induce cell death in various cancer types [38–40]. APR-246 exerts its antitumor activity through apoptosis and autophagy [40, 41]. Besides, p53-independent mechanisms have been also proposed to elucidate the additional effect of APR-246 [41–43]. Among p53-independent pathways, the widely accepted mechanisms are APR-246 which can induce ROS accumulation by depletion of GSH [44, 45]. The cell lines used in this study harbor wild type p53, so our results reveal and confirm the p53-independent functions of APR-246. Despite low ROS levels in cisplatin-resistant cells owing to decreased NADPH metabolism or other mechanism, APR-246 could only lead aberrant ROS only in cisplatin-resistant cells. In details, APR-246 could disrupt ROS-related NRF2/SLC7A11/GSH axis through mediating NRF-2 degradation in cisplatin-resistant cells (Figure 6(g)).

NRF2 is an essential transcription factor that regulates series of detoxifying and antioxidant defense gene expression [36, 46]. It is activated in response to oxidative stress and induces the expression of its target genes by binding to the antioxidant response element (ARE) [47]. We and other groups prove that SLC7A11 is regulated by NRF2 directly [48–50]. Due to NRF2's important functions, regulation of NRF2 is tightly regulated by various mechanisms. Among these, Keap1-NRF2 pathway is the major signaling regulating activity of NRF2, especially after exposure of cytoprotective responses to oxidative and electrophilic stress [51]. As a core repressor protein of NRF2, Keap1 promotes its degradation by the ubiquitin proteasome pathway through direct binding to NRF2 [52–54]. In our study, NRF2 is also specially degraded through ubiquitin proteasome pathway after APR-246 treatment in H460-Cis cells. However, the mechanisms for degradation of NRF2 upon APR-246 treatment need further investigation. Understanding details of this regulatory pathways may contribute to effective pharmacological treatment for cisplatin-resistant NSCLC patients.

Also, NADPH metabolism draws our attention through the entire study. As we all know, NADPH serves as a source of reducing equivalents for the glutathione system. Main functions of NADPH rely on variety of dehydrogenases [55, 56]. We prove that decreased NADPH oxidase-mediated ROS may account for cisplatin resistance of NSCLC based on a published dataset [57]. Besides the maintenance of NADPH, the glutathione metabolism system is also dysregulated after APR-246 treatment. This indicates important roles of NADPH and GSH metabolism in cisplatin resistance. More detailed revelation mechanisms underlying relationships between metabolism and cisplatin resistance would be investigated in our further study.

## 5. Conclusions

In the present study, we identify APR-246 as an effective antitumor compound especially in cisplatin-resistant NSCLC based on our screening panel. APR-246 selectively inhibits proliferation and colony formation of cisplatin-resistant cells instead of parental cells through mitochondria-mediated apoptosis as well as cell cycle dysregulation. Mechanistically, APR-246 could induce ROS production and downregulate NRF2/SLC7A11/GSH axis through mediating NRF-2 degradation in cisplatin-resistant cells. In conclusion, our study shed new light on understanding cisplatin resistance of lung cancer and demonstrate potentials of APR-246 overcoming cisplatin resistance in NSCLC therapy.

## Data Availability

The data used to support the findings of this study are available from the corresponding author upon request.

## Supplementary Materials

Supplementary Materials Figure S1: validation of cisplatin resistance of H460-Cis and A549-Cis cells. Figure S2: concentration- and time-dependent effects of APR-246 in H460-Cis. Figure S3: dysregulation of cell cycle caused by APR-246 in A549-Cis cells. Figure S4: APR-246 leads to mitochondria-mediated apoptosis in A549-Cis cells. Figure S5: APR-246 leads to aberrant ROS in A549-Cis cells. Figure S6: NAC disrupts antitumor effects of APR-246. Table S1: primers used in this study. Table S2: information of antibodies.
