# Supplementary material for: Aberrant ROS Served as an Acquired Vulnerability of Cisplatin-Resistant Lung Cancer
Source: Oxid Med Cell Longev. 2022 Jun 20;2022:1112987. doi: 10.1155/2022/1112987 (PMC9236771; doi:10.1155/2022/1112987)
Supplement: Supplementary Materials — Figure S1: validation of cisplatin resistance of H460-Cis and A549-Cis cells. Figure S2: concentration- and time-dependent effects of APR-246 in H460-Cis. Figure S3: dysregulation of cell cycle caused by APR-246 in A549-Cis cells. Figure S4: APR-246 leads to mitochondria-mediated apoptosis in A549-Cis cells. Figure S5: APR-246 leads to aberrant ROS in A549-Cis cells. Figure S6: NAC disrupts antitumor effects of APR-246. Table S1: primers used in this study. Table S2: information of antibodies. [file 1112987.f1.zip › 1112987.f1/Supplemental Figures_2022-05-22-Final.docx]

**
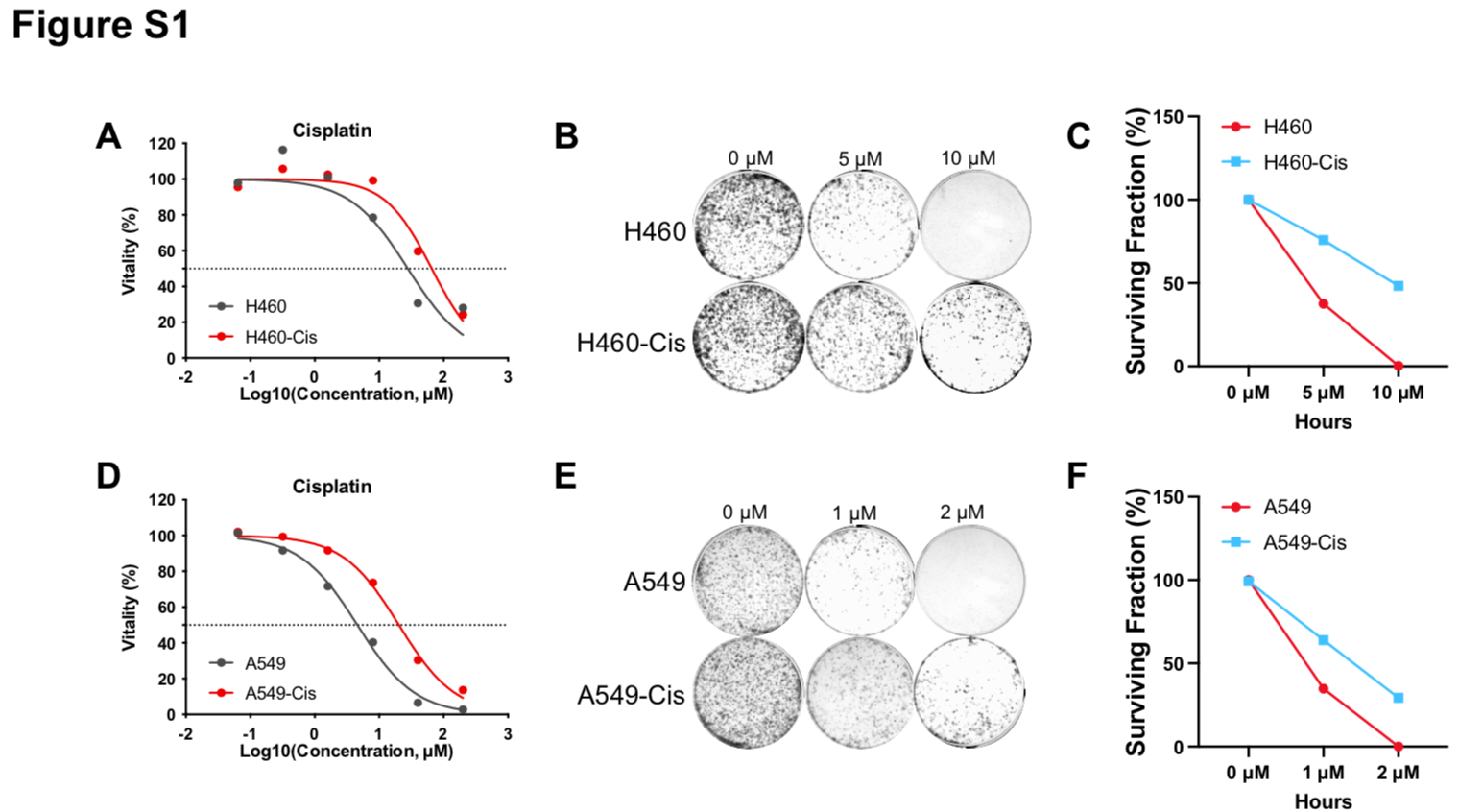
**

**Figure S1. Validation of cisplatin resistance of H460-Cis and A549-Cis cells**

(A) Dose response curves for cisplatin of H460 and H460-Cis cells.

(B-C) Colony formation and surviving fractions of H460 and H460-Cis cells treated with internal and high dose cisplatin.

(D) Dose response curves after treatment with cisplatin in A549 and A549-Cis cells.

(E-F) Colony formation and survival fractions for A549 and A549-Cis cells treated with different dose cisplatin.


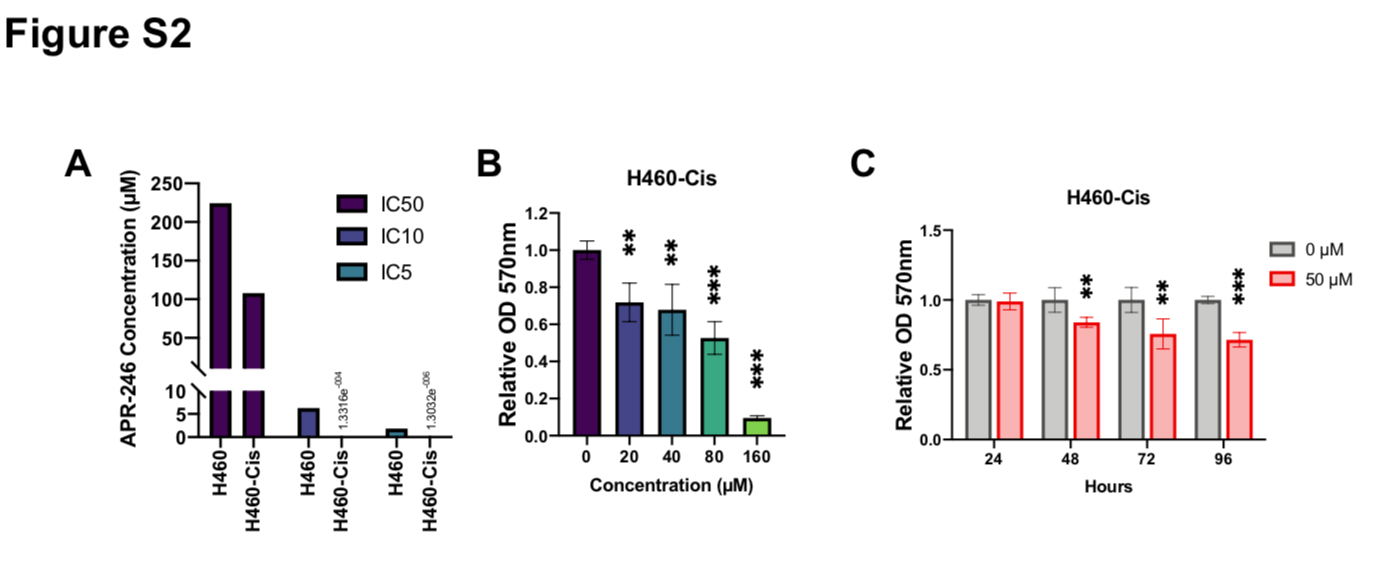


**Figure S2. Concentration- and time- dependent effects of APR-246 in H460-Cis**

(A) IC5, IC10 and IC50 values of APR-246 in both H460 and H460-Cis cells.

(B) Dose dependent anti-tumor effects of APR-246 in H460-Cis cells.

(C) The effectiveness of time dependent anti-tumor of APR-246 in H460-Cis cells.

**p < 0.01; ***p < 0.001 (two-tailed unpaired t-test).


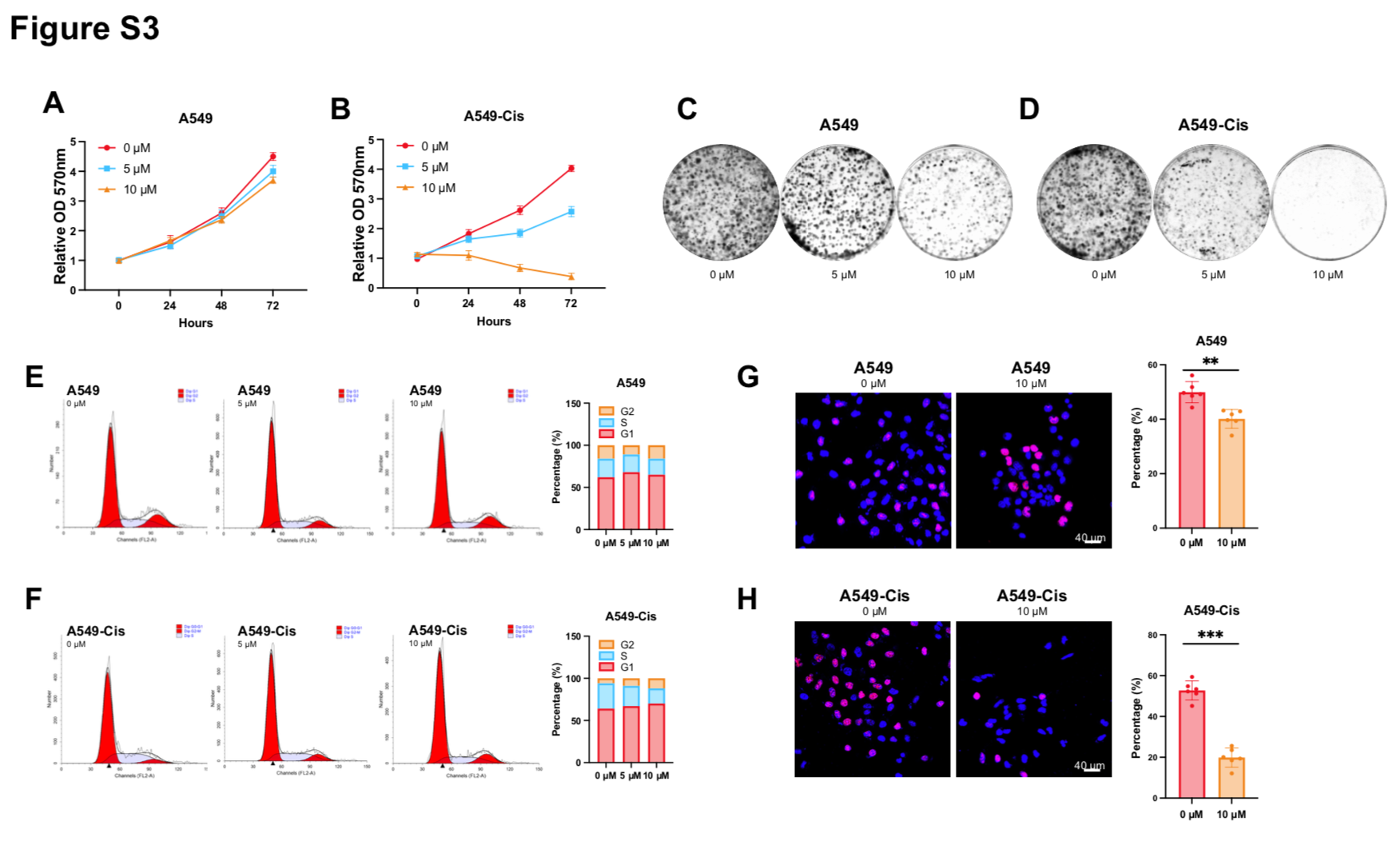


**Figure S3. Dysregulation of cell cycle caused by APR-246 in A549-Cis cells**

(A-B) Growth curves of A549 and A549-Cis cells treated with APR-246 and control group.

(C-D) Colony formation assays of A549 and A549-Cis cells with APR-246 treatment or not.

(E-F) Cell cycle distribution of A549 and A549-Cis cells treated with APR-246 or not.

(G-H) Results of EdU incorporation assay detected in APR-246 treating A549 and A549-Cis cells.

Error bars represent SD of three independent experiments.

**p < 0.01; ***p < 0.001 (two-tailed unpaired t-test).


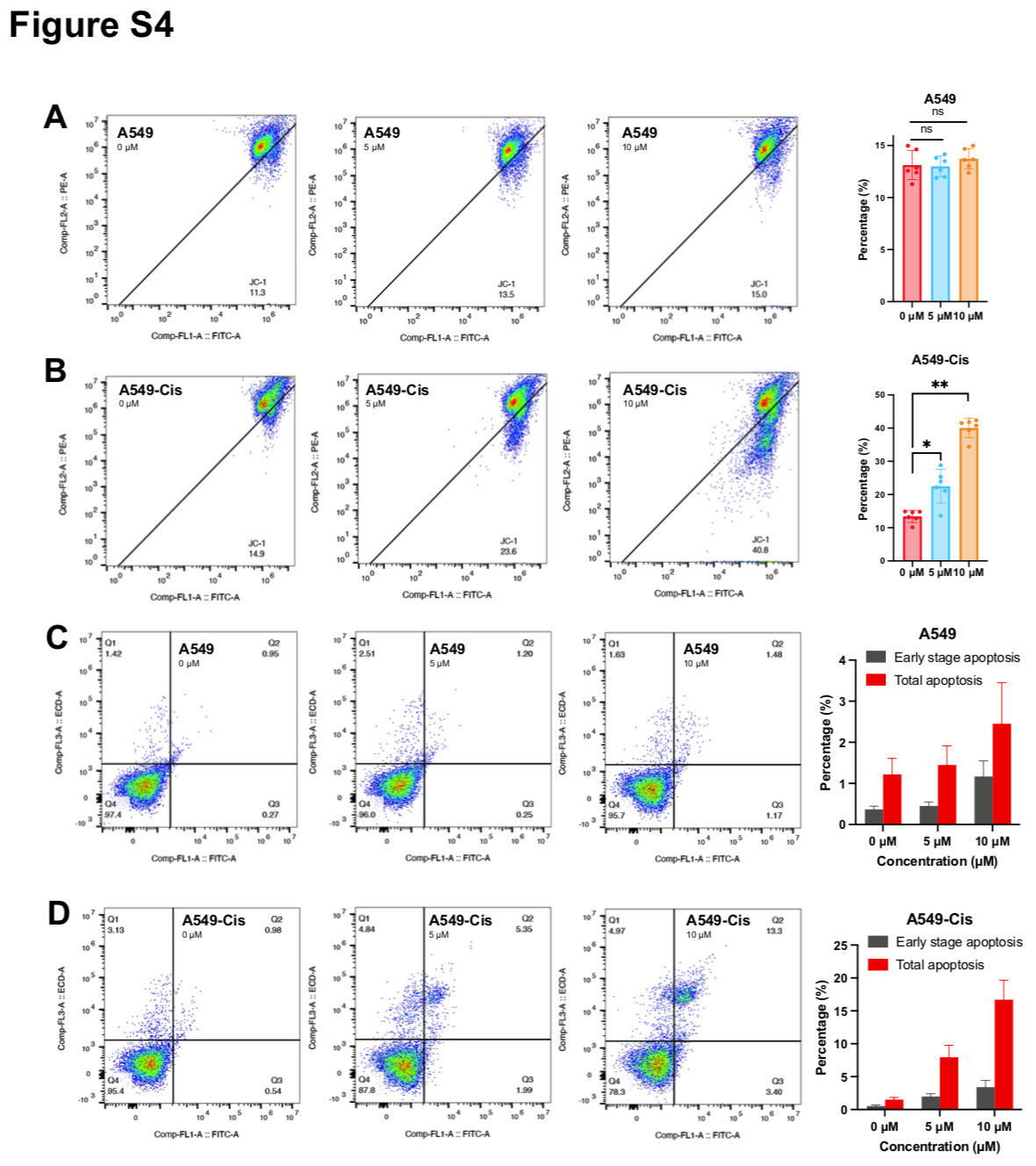


**Figure S4. APR-246 leads to mitochondria mediated apoptosis in A549-Cis cells**

(A-B) Levels of mitochondrial membrane potentials of A549 and A549-Cis cells with APR-246 treatment are detected by JC-1 assay. Representative results are shown on the left and statistical analysis is on the right.

(C-D) Apoptosis results of A549 and A549-Cis cells treated with APR-246 or not. Representative apoptosis results are shown on the left and statistical analysis is on the right side.

*p < 0.05; **p < 0.01; ns, not statistically significant (two-tailed unpaired t-test).


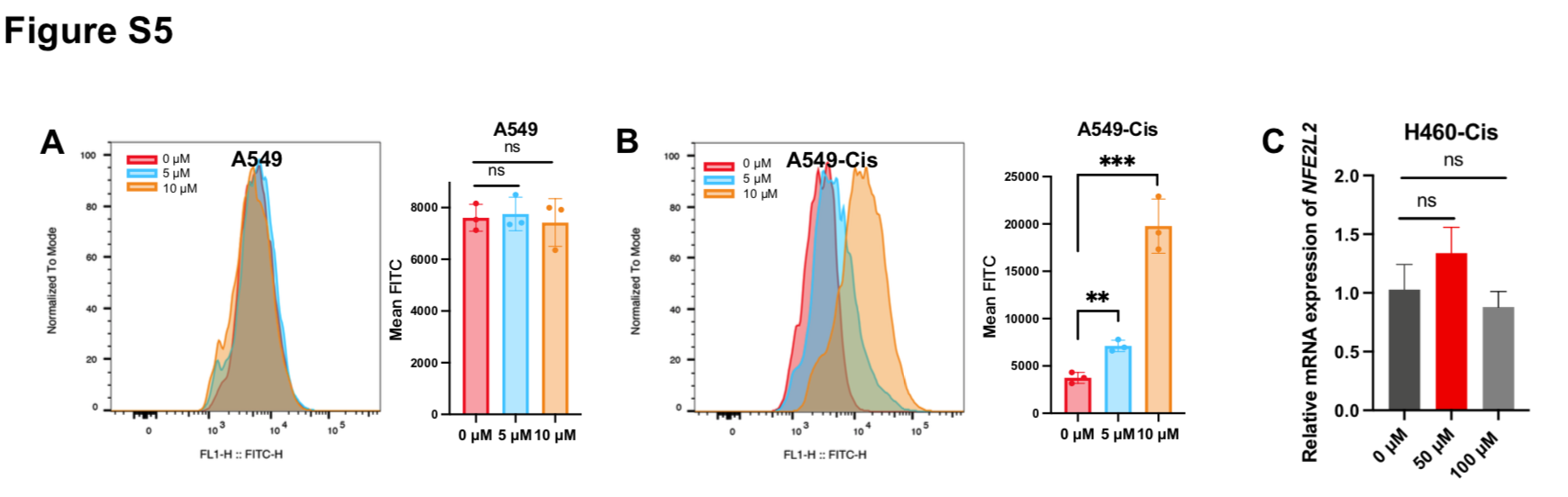


**Figure S5. APR-246 leads to Aberrant ROS in A549-Cis cells**

(A-B) Detection of ROS levels in A549 and A549-Cis cells treated with APR-246 by FCM. Representative results are shown on the left and statistical analysis for each group is shown on the right.

(C) Expression levels of *NFE2L2* are determined by Real-time RT-PCR in H460-Cis cells treated with APR-246 or not.

**p < 0.01; ***p < 0.001; ns, not statistically significant.


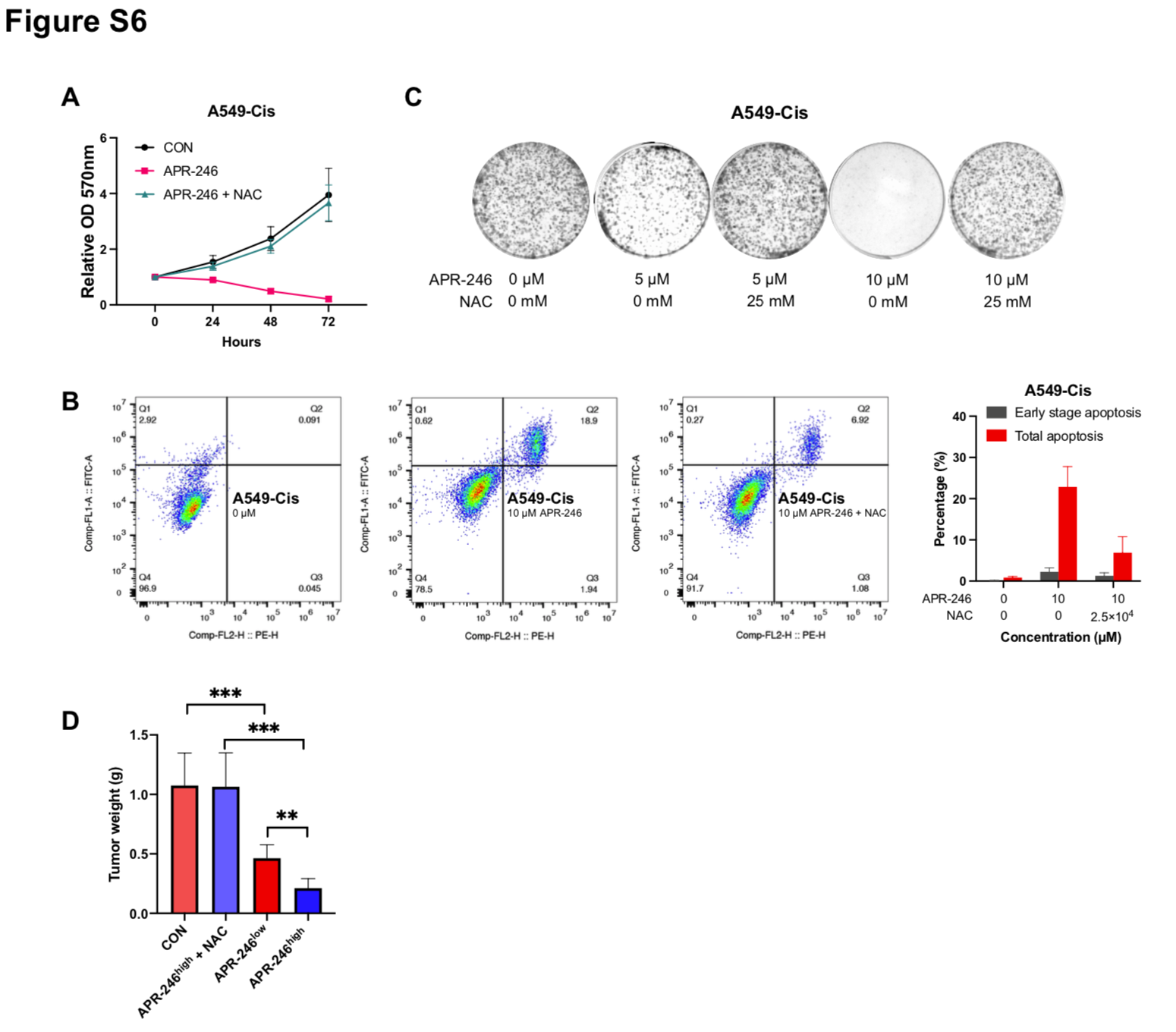


**Figure S6. NAC disrupts anti-tumor effects of APR-246**

(A) Cell proliferation of A549-Cis cells treated with APR-246 or/and NAC.

(B) Apoptosis levels of A549-Cis cells were measured by FCM after treatment with APR-246 or/and NAC. The right panel stands for statistical analysis results.

(C) Colony formation ability of A549-Cis cells treated with APR-246 or/and NAC.

(D) Tumor weight of Xenograft of H460-Cis cells treated with APR-246 or/and NAC.

**p < 0.01; ***p < 0.001 (two-tailed unpaired t-test).
