# Supplementary material for: Aberrant ROS Served as an Acquired Vulnerability of Cisplatin-Resistant Lung Cancer
Source: Oxid Med Cell Longev. 2022 Jun 20;2022:1112987. doi: 10.1155/2022/1112987 (PMC9236771; doi:10.1155/2022/1112987)
Supplement: Supplementary Materials — Figure S1: validation of cisplatin resistance of H460-Cis and A549-Cis cells. Figure S2: concentration- and time-dependent effects of APR-246 in H460-Cis. Figure S3: dysregulation of cell cycle caused by APR-246 in A549-Cis cells. Figure S4: APR-246 leads to mitochondria-mediated apoptosis in A549-Cis cells. Figure S5: APR-246 leads to aberrant ROS in A549-Cis cells. Figure S6: NAC disrupts antitumor effects of APR-246. Table S1: primers used in this study. Table S2: information of antibodies. [file 1112987.f1.zip › 1112987.f1/Supplemental Table 1-Primer sequences.docx]

**Supplemental Table 1. Primer sequences**

| Name | Sequence |
| --- | --- |
| *SLC7A11*-qF | ACTCCTCATAATACGCCCTG |
| *SLC7A11*-qR | CCATCACTACAGTTATGCCCA |
| *NFE2L2*-qF | CAGCACATCCAGTCAGAAACC |
| *NFE2L2*-qR | GTAGCCGAAGAAACCTCATTGTC |
| *GAPDH*-q-F | ATTCTCTGATTTGGTCGTATTGGG |
| *GAPDH*-q-R | ATGACAAGCTTCCCGTTCTC |
