# Supplementary material for: Aberrant ROS Served as an Acquired Vulnerability of Cisplatin-Resistant Lung Cancer
Source: Oxid Med Cell Longev. 2022 Jun 20;2022:1112987. doi: 10.1155/2022/1112987 (PMC9236771; doi:10.1155/2022/1112987)
Supplement: Supplementary Materials — Figure S1: validation of cisplatin resistance of H460-Cis and A549-Cis cells. Figure S2: concentration- and time-dependent effects of APR-246 in H460-Cis. Figure S3: dysregulation of cell cycle caused by APR-246 in A549-Cis cells. Figure S4: APR-246 leads to mitochondria-mediated apoptosis in A549-Cis cells. Figure S5: APR-246 leads to aberrant ROS in A549-Cis cells. Figure S6: NAC disrupts antitumor effects of APR-246. Table S1: primers used in this study. Table S2: information of antibodies. [file 1112987.f1.zip › 1112987.f1/Supplemental Table 2- Information of antibodies.docx]

**Supplemental Table 2. Information of antibodies**

| Name | Company | Catalog Number |
| --- | --- | --- |
| PARP | Cell Signaling Technology | 9542 |
| Cleaved PARP | Cell Signaling Technology | 5625 |
| Caspase-3 | Cell Signaling Technology | 14220 |
| Cleaved Caspase-3 | Cell Signaling Technology | 9664 |
| Caspase-7 | Cell Signaling Technology | 12827 |
| Cleaved Caspase-7 | Cell Signaling Technology | 8438 |
| Caspase-9 | Cell Signaling Technology | 9508 |
| Cleaved Caspase-9 | Cell Signaling Technology | 52873 |
| SLC7A11 | Abcam | ab175186 |
| NRF2 | Abcam | ab62352 |
| β-Actin | Proteintech | 20536-1-AP |
